# Involvement of NFƙB and MAPK signaling pathways in the preventive effects of *Ganoderma lucidum* on the inflammation of BV-2 microglial cells induced by LPS

**DOI:** 10.1016/j.jneuroim.2020.577269

**Published:** 2020-05-26

**Authors:** Aaron Hilliard, Patricia Mendonca, Karam F.A. Soliman

**Affiliations:** Division of Pharmaceutical Sciences, College of Pharmacy and Pharmaceutical Sciences, Florida A&M University, Tallahassee, FL 32307, United States of America

**Keywords:** *Ganoderma lucidum*, Alzheimer’s disease, Inflammatory cytokines, MIP3α, *NFKB1/p50*, *IKBKE*

## Abstract

*Ganoderma lucidum* extract (GLE) is a potent ancient Asian remedy for the treatment of various diseases. This study investigated GLE preventive effects on LPS-stimulated inflammation of BV-2 microglial cells. The results show that pre-treatment with GLE decreased expression of pro-inflammatory cytokines: G-CSF, IL1-α, MCP-5, MIP3α, and, with a higher effect in MIP3α. In RT-PCR assays, pre-treatment with GLE decreased mRNA expression of *CHUK*, *NFκB1/p150*, and *IKBKE* (NFƙB signaling), which may be associated with the neuropathology of Alzheimer’s disease. The data show GLE inhibiting ability on pro-inflammatory mediators’ release and suggest a potential role of GLE in neurodegenerative disease prevention.

## 1. Introduction

Inflammation is a hallmark of many neurodegenerative diseases, and the microglia, the resident macrophages, regulate immunity in the CNS (Gonzalez-Scarano and Baltuch, 1999; Schwab and Schluesener, 2004; Kim and de Vellis, 2005; Block et al., 2007). The innate immune system stimulates the development of inflammation to protect the brain and its neurons (Lefkowitz and Lefkowitz, 2008; Ransohoff and Perry, 2009). The acute activation of the microglia is a neuroprotective process and leads to the elevation of pro-inflammatory cytokines (Kim et al., 2019b; Kim et al., 2019a). Moreover, the existence of chronic inflammation may lead to the overproduction of pro-inflammatory cytokines, which play a significant role in neurodegenerative diseases such as Alzheimer’s disease (AD) and Parkinson’s disease (PD) (Akiyama et al., 2000; Bamberger et al., 2003; Sheng et al., 2003; Streit et al., 2004; Rojo et al., 2008; Cobourne-Duval et al., 2018).

Moreover, the activation of MAP kinase (MAPK) and nuclear factor kappa B (NFƙB) pathways is known to be associated with the release of critical cytokines related to inflammation. One of the first responses to stress is cell signaling from the cytosol to the nucleus, which leads to the production of pro-inflammatory cytokines and chemokines involved in a dose-dependent activation of in NFƙB. The exact mechanism involved in neuronal injury in AD and PD is not fully understood. Also, in AD patients, amyloid-beta (Aβ) plaques are distinguishing hallmarks of this disease state. These plaques have been shown to activate gene expression of NFƙB, which leads to the translocation of pro-inflammatory cytokines outside of the cell (Tilstra et al., 2011; Mendonca et al., 2018). The release of these cytokines is critical to the induction and maintenance of neural inflammation (Mendonca et al., 2018). As chronic neuroinflammation is involved as a crucial factor in the pathology of neurodegenerative diseases, the need for exploring anti-inflammatory and preventive therapies is warranted.

Certain mushrooms have not only nutritional value but also contain bioactive compounds with high medicinal properties. One such mushroom, the *Ganoderma lucidum*, is used across Asia as a nutraceutical and herbal remedy for many different ailments (Ding et al., 2010; Batra et al., 2013; Collado Mateo et al., 2015; Wang et al., 2018a; Geng et al., 2019). *Ganoderma lucidum* extract (GLE) has shown therapeutic benefits as an anti-diabetic agent, in breast cancer, and in inflammatory diseases such as colitis (Martinez-Montemayor et al., 2011; Suarez-Arroyo et al., 2013; Liu et al., 2015; Barbieri et al., 2017; Geng et al., 2019; Liu and Tie, 2019; Yin et al., 2019). Studies have also suggested that GLE has a neuroprotective effect and may be a useful therapeutic option for the prevention of AD and PD (Cheung et al., 2000; Zhao et al., 2004; Chen et al., 2007; Lai et al., 2008; Zhou et al., 2010; Shen et al., 2013; Yoon et al., 2013; Wang et al., 2018b).

In a recent study, deacetyl ganoderic acid F (DeGA F), which is a triterpenoid isolated from *G lucidum, was tested* in activated-BV-2 microglial cells. DeGA F inhibited mRNA expression of *TNF-α*, *IL-6*, and *IL-1β*. Also, the pretreatment with DeGA F inhibited the protein expression of Akt, IKKα/β, and IκBα (which participate in the NFƙB activation), compared to the LPS-stimulated cells (Sheng et al., 2019). Polysaccharides from *G. lucidum* have also been shown to inhibit LPS- and Aβ-induced pro-inflammatory levels of IL-1β, IL-6, and iNOS, and induce the expression of the anti-inflammatory cytokine TGFβ (Cai et al., 2017). However, the specific mechanisms underlying the anti-inflammatory nature of GLE have not been fully explored.

In the present study, we examined the preventive effects of *G. lucidum* fruiting body dry extract (United States Pharmacopeia (*USP*) standard), on LPS-activation of microglia BV-2 cells. The goal was to screen 120 cytokines and investigate whether GLE had the ability to downregulate pro-inflammatory cytokines that could be used as therapeutic targets in the prevention and slowing the progression of neurodegenerative diseases, where chronic inflammation plays a crucial role in the disease pathogenesis. Also, we studied GLE’s ability to modulate genes associated with NFƙB, MAPK, and nucleotide-binding oligomerization domain (NOD) signaling pathways, which are known to have a crucial role in inflammatory processes through their capability to induce transcription of pro-inflammatory genes. Therefore, the current investigation is designed to test the hypothesis that a preventive effect of GLE on inflammation is mediated through its ability to attenuate NFƙB and MAPK signaling pathways of the microglial cells.

## 2. Materials and methods

### 2.1. BV-2 microglial cell model

We used the well-characterized immortalized microglial BV-2 cell line in this study. BV-2 cell line is well-suited to study the microglia role in modulating chronic inflammation observed in neurodegenerative disorders, such as AD and PD (Sarkar et al., 2018). This microglial cell is an efficient model to study inflammation, and it has been used in about 75% of publications to investigate the biology of microglial cells as well as their role in neuroinflammation (Sarkar et al., 2018). Moreover, studies comparing primary microglia (PM) and BV-2 cells demonstrated that BV-2 cells have an overall response pattern that parallels that of PM. About 90% of the genes that are regulated in BV-2 cells are also found in PM (Henn et al., 2009). Thus, this model is suitable to investigate the preventive effects of GLE on the LPS-activation of BV-2 microglial cells.

### 2.2. Ganoderma lucidum extract

Taking in consideration the pharmacological potential of *G. lucidum*, the fact that the GLEs described in the literature have different phytochemical constituents, and that the specific mechanisms underlying its anti-inflammatory nature have not been fully explored yet, in this study we investigated the effect of a *G. lucidum* extract that was obtained from Sigma-Aldrich (GLE Cat# 1288372)/ U.S. Pharmacopeial Convention on *Ganoderma Lucidum* fruiting body dry extract. The extract is a mixture of ganoderenic acid C, ganoderic acid C2, ganoderic acid G, ganoderenic acid B, ganoderic acid B, ganoderic acid A, ganoderic acid H, ganoderenic acid D, ganoderic acid D, ganoderic acid F, and polysaccharides (USP Cat # 1288372 - USP lot # F012B0).

### 2.3. Reagents

Alamar Blue® powder was obtained from Sigma-Aldrich Co. (St. Louis, MO). Dulbecco’s modified Eagle’s medium (DMEM) high glucose, fetal bovine serum heat-inactivated (FBS-HI), and penicillin/streptomycin were obtained from Genesee Scientific (San Diego, CA, USA). Mouse cytokine antibody arrays (cat# AAM-CYT-1000) and ELISA assays were purchased from RayBiotech (Norcross, Ga, USA). PCR primers, iScript advanced reverse transcriptase kit, and SYBR Green were purchased from Bio-Rad (Hercules, CA, USA). Turbo DNA-free™ Kit (Cat# AM1907) from Life Technologies Inc. (Grand Island, NY, USA).

### 2.4. Cell culture

BV-2 microglial cells were kindly provided by Dr. Elizabeta Blasi (Blasi et al., 1990) and were cultured as previously described (Mendonca et al., 2017; Mendonca et al., 2018). Briefly, cells were grown in DMEM media supplemented with 10% heat-inactivated fetal bovine serum and 1% penicillin/streptomycin (100 U/ml penicillin and 0.1 mg/ml streptomycin). Cultures were incubated in a humidified atmosphere of 5% CO_2_ at 37 °C.

### 2.5. Cell viability

BV-2 cell viability was assessed using Alamar Blue® (Resazurin) assay. Cells were plated at approximately 3 × 10^5^ cells/ml (100 μl/well) in 96-well plates and were incubated overnight to allow attachment to the substrate. The next day, cells were replaced with experimental media containing treatment with different concentrations of GLE, ranging from 0.5 to 1.3 mg/ml, and incubated at 37 °C for 1 h. Cells were then activated with LPS (1.0 μg/ml). BV-2 control cells were treated with experimental media that received only dH_2_0, which was used to dissolve GLE extract. After 24 h’ incubation, 20 μl of Alamar Blue® was added, and cells were incubated again for 4 h. Cell viability was determined using a microplate reader (Infinite M200, Tecan Trading AG). This assay generated a fluorescence spectrophotometrically measured at 550 nm excitation and 580 nm emission wavelengths, which is proportional to the number of viable cells. Data were expressed as a percentage of live, untreated control cells.

### 2.6. Measurement of nitric oxide (NO) production

Nitric oxide production was measured in activated BV-2 cells in the presence of GLE. BV-2 cells (3 × 10^4^ cells/well in a 96-well plate) were incubated overnight to allow attachment to the substrate. Cells were pre-treated with GLE (concentration range from 0.0125 to 0.6 mg/ml), activated with LPS after 1 h, and then incubated again for 24 h. Control cells were treated with dH_2_0. Nitric oxide production in cell supernatant was evaluated spectrophotometrically by measuring the amount of nitrite produced, which is the oxidative product of nitric oxide. Equal amounts of cell supernatant (50 μl) and Griess reagent (Subedi et al., 2016) were mixed, and OD was measured at 550 nm. The sodium nitrite standard curve was used to determine nitrite concentrations in the supernatant.

### 2.7. Mouse cytokine antibody arrays

Cytokine antibody arrays (RayBiotech mouse cytokine antibody arrays, Cat# AAM-CYT-1000) were used to study the effect of GLE on 120 cytokine protein expression levels released by BV-2 microglial cells stimulated by LPS. Each experiment was performed in triplicate according to the manufacturer’s instructions and as previously described (Mendonca et al., 2017). Briefly, antibody-coated array membranes were incubated for 30 min with 1 ml of blocking buffer. Blocking buffer was then decanted and replaced with 1 ml supernatant from control (dH_2_O only) samples, cells treated with GLE (0.5 mg/ml) only, LPS only (1.0 μg/ml), and GLE (0.5 mg/ml) + LPS (1.0 μg/ml), where LPS was added after 1 h incubation with GLE. Membranes were incubated overnight at 4 °C with mild shaking. Media were decanted the next day; membranes were washed, and subsequently incubated with 1 ml biotin-conjugated antibodies for 2 h. Biotin-conjugated antibodies were removed, and membranes were incubated with HRP-conjugated streptavidin overnight. The next day, detection of spots was acquired using chemiluminescence with semi-quantitative analysis of signal intensities from Quantity One software (Bio-Rad). Intensities were normalized as a percentage of positive controls on each membrane.

### 2.8. Cytokine ELISAs

BV-2 cells were treated for 24 h and then supernatants from controls (dH_2_O only), from cells treated with GLE (0.5 mg/ml) only, LPS only (1.0 μg/ml), and GLE (0.5 mg/ml) + LPS (1.0 μg/ml) (where LPS was added after 1 h incubation with GLE), were used in the assay. Specific ELISAs (RayBiotech, Norcross, GA, USA) were performed using G-CSF (Cat# ELM-G-CSF), IL1α (Cat# ELM-IL1a), MCP-5 (Cat# ELM-MCP5), MIP3α (Cat# ELM-MIP3a), and RANTES (Cat# ELM-RANTES) following manufacturer’s instructions and as previously described (Mendonca et al., 2017). Briefly, 100 μl of supernatant from samples and standards were added to 96 well plates pre-coated with the capture antibody. After incubation, 100 μl of prepared biotinylated antibody mixture was added to each well and incubated for 1 h. The mixture was then decanted, and streptavidin solution (100 μl) was placed in each well and incubated. Substrate reagent (100 μl) was then added to each well for 30 min, followed by the addition of stop solution (50 μl). Data were quantified by optical density at 450 nm.

### 2.9. Real time-polymerase chain reaction (RT-PCR)

#### 2.9.1. RNA extraction, cDNA synthesis, and RT-PCR

BV-2 cells were treated dH_2_O only (control), GLE (0.5 mg/ml) only, LPS only (1.0 μg/ml), and GLE (0.5 mg/ml) + LPS (1.0 μg/ml) (where LPS was added after 1 h incubation with GLE), and incubated for 24 h. The cells were then harvested and washed twice with PBS. Cell pellets were lysed using 1 ml Trizol reagent. Chloroform (0.2 ml) was added to the samples; the tubes were shaken, incubated at 15–30 °C for 2–3 min and centrifuged at 10,000*g* for 15 min at 2–8 °C. The aqueous phase was transferred to a fresh tube, and the RNA precipitated by mixing 0.5 ml of isopropyl alcohol. Samples were then centrifuged, the supernatant removed, and the RNA pellets washed with 75% ethanol. Samples were centrifuged at 7500g for 5 min at 2–8 °C; the RNA pellet was dried and dissolved in RNase-free water and incubated for 30 min on ice before use. RNA purity and quantity were determined using Nanodrop (Thermo Fischer Scientific - Wilmington, DE, USA). The cDNA strand was synthesized using iScript advanced reverse transcriptase (Bio-Rad – Hercules, CA). A solution of 4 μl of the 5× iScript advanced reaction mix (containing primers), 1 μl of reverse transcriptase, 7.5 μl of the sample (1.5 μg/7.5 μl) for RT-PCR, and 7.5 μl water were added to 0.2 ml tubes, in a total of 20 μl. The thermal cycling program for reverse transcription included two steps: 42 °C for 30 min and then 85 °C for 5 min. Real-time PCR amplification was performed following the Bio-Rad protocol. A 1 μl of the sample (200 ng cDNA/reaction), 10 μl of the master mix, 1 μl of primer, and 8 μl of water were added to each tube for the individual RT-PCRs. The thermal cycling process including the initial hold step at 95 °C for 2 min and denaturation at 95 °C for 5 s, followed by 40 cycles of 60 °C for 30 s (annealing/extension), and 60 °C for 5 s/step (melting curve) using Bio-Rad CFX96 Real-Time System (Hercules, CA, USA.

The Unique Assay ID for each primer is described as follows: *CHUK* (UniqueAssayID: qMmuCID0005344); *IKBKE* (UniqueAssayID: qMmuCID0022200; *IRAK1* (UniqueAssayID: qMmuCID0005129); *NFƙB1* (UniqueAssayID: qMmuCID0005357); *NOD1* (UniqueAssayID: qMmuCID0015768).

## 3. Data analysis

Statistical analysis was performed using Graph Pad Prism (version 6.07). All data points are expressed as the mean ± S.E.M. from at least 3 independent experiments. For the viability studies, the IC_50_ was determined by nonlinear regression with R^2^ best fit and lowest 95% confidence interval. Statistically significant differences between different groups in the experiments were assessed using a one-way ANOVA, followed by Dunnett’s multiple comparison tests (*P < .05, **P < .01, ***P < .001, ****P < .0001, and ns = *p* > .05). Gene expression was analyzed using the CFX 3.1 Manager software (Bio-Rad, Hercules, CA).

## 4. Results

**Fig. 1.**
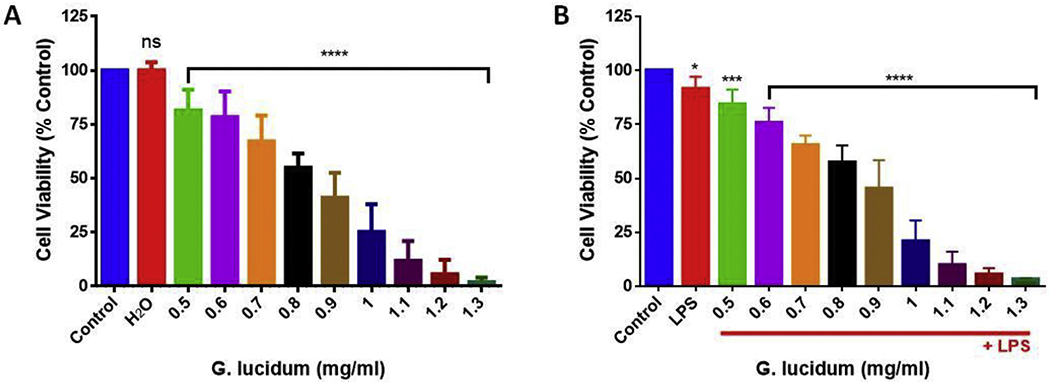
Dose-response decrease in cell viability by GLE in BV-2 microglia cells. GLE tested concentrations ranged from 0.5 to 1.3 mg/ml. All experiments were performed at least 3 times (*n* = 5) and kept at 5% CO_2_ and 37 °C. The cytotoxic effect was measured after 24 h using Alamar Blue®. (A) Cells were treated with different concentrations of GLE. (B) Cells were pre-treated with different concentrations of GLE and stimulated with LPS (1 μg/ml) after 1 h. The data are presented as the mean ± S.E.M. Statistically significant differences between control vs. treatments were evaluated by a one-way ANOVA, followed by Dunnett’s multiple comparison tests. *p < .05, ***p < .001, ****p < .0001, ns = *p* > .05. (For interpretation of the references to colour in this figure legend, the reader is referred to the web version of this article.)

Cell viability was assessed in BV-2 microglial cells treated with GLE only and a combination of GLE and LPS (after 1 h). We observed a dose-dependent decrease in cell viability in concentrations of GLE at and above 0.5 mg/ml (Fig. 1A). The combination of 0.5 mg/ml of GLE and 1.0 μg/ml of LPS did not change the IC_50_ of 0.79 mg/ml observed when only GLE was used (Fig. 1B). Based on these results, 0.5 mg/ml of GLE and 1.0 μg/ml of LPS were chosen as our working concentrations in subsequent experiments.

**Fig. 2.**
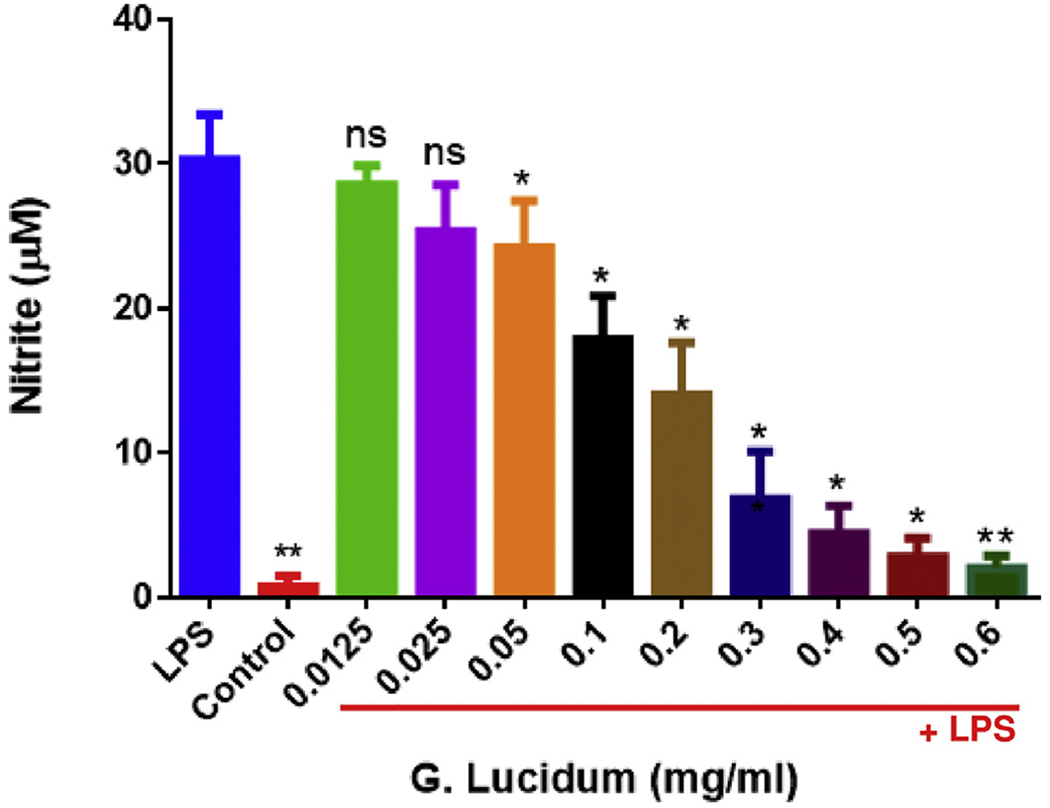
Dose-response decrease in nitric oxide production by GLE pre-treatment in BV-2 microglial cells stimulated by LPS. The concentrations of GLE ranged from 0.0125 to 0.6 mg/ml. All experiments were performed at least 3 times (n = 5) at 5% CO_2_ and 37 °C for 24 h. Cells were pre-treated with GLE and after 1 h stimulated with LPS (1 μg/ml). The amount of nitrite was measured using Griess reagent. The data are presented as mean ± S.E.M Statistically significant differences between LPS vs. GLE + LPS-treatments were evaluated by a one-way ANOVA, followed by Dunnett’s multiple comparison tests. *p < .05, **p < .01, ns = p > .05.

The effect of GLE on nitrite production in BV-2 microglial cells was examined in cells that were pre-treated with the compound for 1 h and then activated with LPS (1 μg/ml) for 24 h. Nitrite production was increased by 30-fold when cells were stimulated with LPS, compared to control. Neither 0.0125 mg/ml nor 0.025 mg/ml of GLE caused inhibition of nitrite in the LPS-stimulated cells. However, GLE showed a significant dose-dependent decrease in nitrite production in concentrations of 0.05 through 0.6 mg/ml, indicating GLE capability to significantly reduce nitrite production during acute inflammation (Fig. 2).

**Fig. 3.**
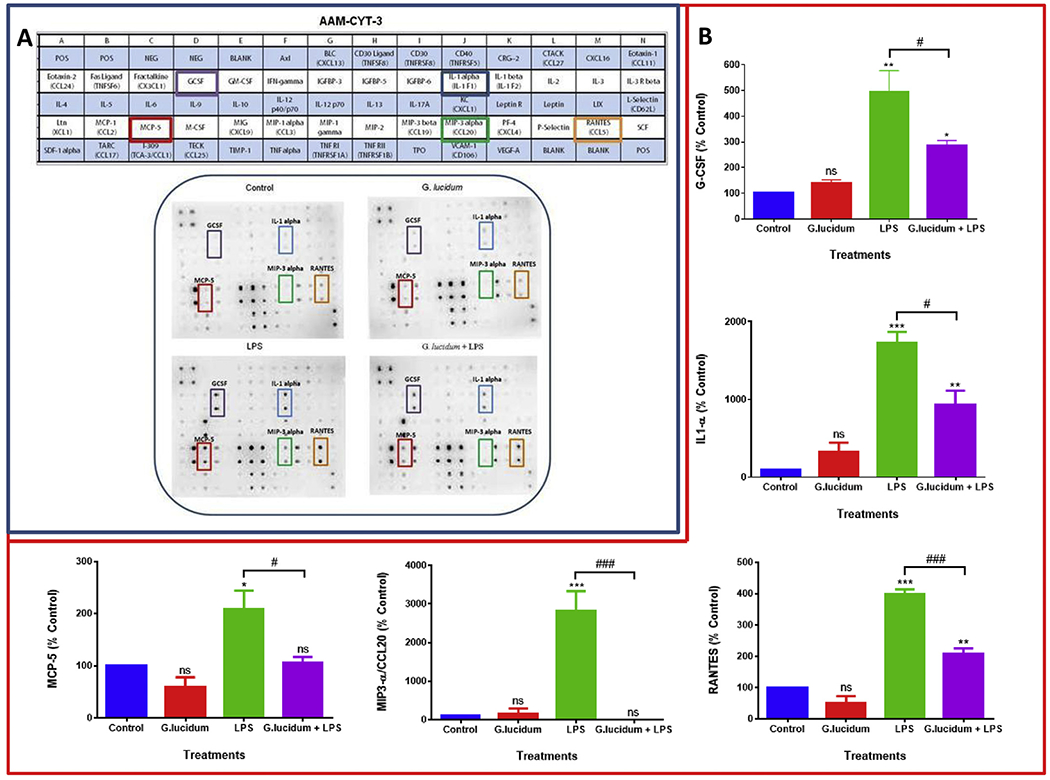
Inhibition of cytokines expression by GLE pre-treatment in BV-2 microglial cells stimulated by LPS. A- Array layout used to assess chemokines/cytokines expression in the cell-free supernatants, highlighting the proteins downregulated by GLE and chemiluminescent spot intensity of supernatants derived from BV-2 cells showing cytokine changed expression after treatments. B- Graphs represent normalized protein expression of G-CSF, IL1α, MCP-5, MIP3α, and RANTES modulated by different treatments in BV-2 cells. Data are expressed as % of control arrays (mean ± S.E.M.) and correspond to normalized dot spot intensities from the cytokine arrays calculated based on the positive controls found in the corners of each one of the membranes using RAYBIO®ANALYSIS software. Blots and graphs represent the supernatants of: control (cells + dH_2_O), GLE (0.5 mg/ml), LPS (1 μg/ml), and GLE (0.5 mg/ml) + LPS (1 μg/ml) after 1 h, in a 24-h treatment period (*n* = 3). Statistically significant differences between control vs. treatments (*), and LPS vs. GLE + LPS (^#^) were evaluated by a one-way ANOVA, followed by Dunnett’s multiple comparison tests. *p < .05, **p < .01, ***p < .001, ^#^p < .05, ^###^p < .01, ns = p > .05.

**Fig. 4.**
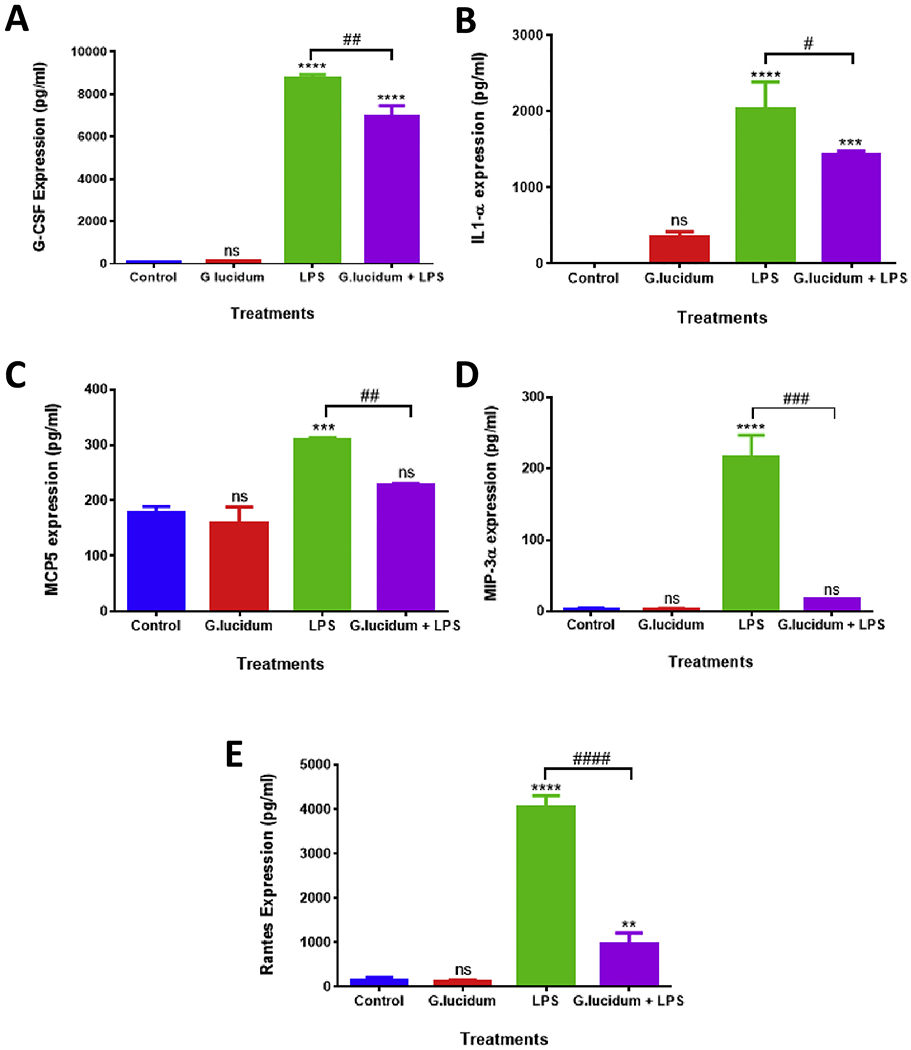
Inhibition of protein expression of G-CSF, IL1α, MCP-5, MIP3α, and RANTES by GLE pre-treatment in BV-2 cells stimulated by LPS, using ELISA assay. The effect of the pre-treatment with GLE on G-CSF, IL1α, MCP-5, MIP3α, and RANTES protein expression in BV-2 cells stimulated by LPS was investigated with individual ELISAs. Each data point represents the mean ± S.E.M. of three independent experiments (n = 3), representing 4 treatments: control (cells + dH_2_O), GLE (0.5 mg/ml) only, LPS (1 μg/ml) only, and GLE (0.5 mg/ml) + LPS (1 μg/ml) after 1 h, in a 24-h treatment period. Statistically significant differences between control vs treatments (*), and LPS vs GLE + LPS (^#^) were evaluated by a one-way ANOVA, followed by Dunnett’s multiple comparison tests. **p < .01, ***p < .001, ****p < .0001, ^#^p < .05, ^##^p < .01, ^###^p < .001, ^####^p < .0001, ns = p > .05.

To determine the effect of GLE, LPS, and GLE + LPS vs. control on the expression profile of pro-inflammatory cytokines, mouse cytokine antibody arrays were used (Fig. 3A). Microarray chemiluminescent spot intensity analysis of supernatants derived from control vs. LPS-stimulated BV-2 microglial cells showed that LPS induced the expression of CXCL1, G-CSF, IL1α, IL-6, MCP-5, MIP3α, and RANTES. The spot intensity was reduced when the supernatants from GLE + LPS-stimulated vs. LPS-stimulated cells were compared, indicating the downregulation of some of these cytokines’ expression (Fig. 3A). Cytokine normalized expression was calculated based on the spot intensity of positive controls in each one of the membranes. LPS significantly (*p* < .05) increased the expression of CXCL1, G-CSF, IL1α, IL-6, MCP-5, MIP3α, and RANTES with a percentage change ranging from 200 to 2800, where MIP3α presented the highest expression. After GLE treatment for 24 h, the cells activated by LPS exhibited a significant inhibition in the production of G-CSF, IL1α, MCP-5, MIP3α, and RANTES (Fig. 3B), but not in CXCL1 and IL-6 (data not shown). While all the cited cytokines were reduced by a 2-fold-change, MIP3α exhibited the most significant reduction, being entirely suppressed by GLE extract (Fig. 3B). Individual ELISA quantitative assays were performed to validate the cytokine array findings. The results confirmed that LPS significantly (*p* < .01) increases the expression of G-CSF, IL1α, MCP-5, MIP3α, and RANTES (Fig. 4A–E). Corroborating the findings of the cytokine arrays, GLE pre-treatment was able to downregulate the expression of these proteins, presenting a higher inhibition in MIP3α (Fig. 4D) and RANTES (Fig. 4E), with a decreased fold-change of 13 and 4.2, respectively.

**Fig. 5.**
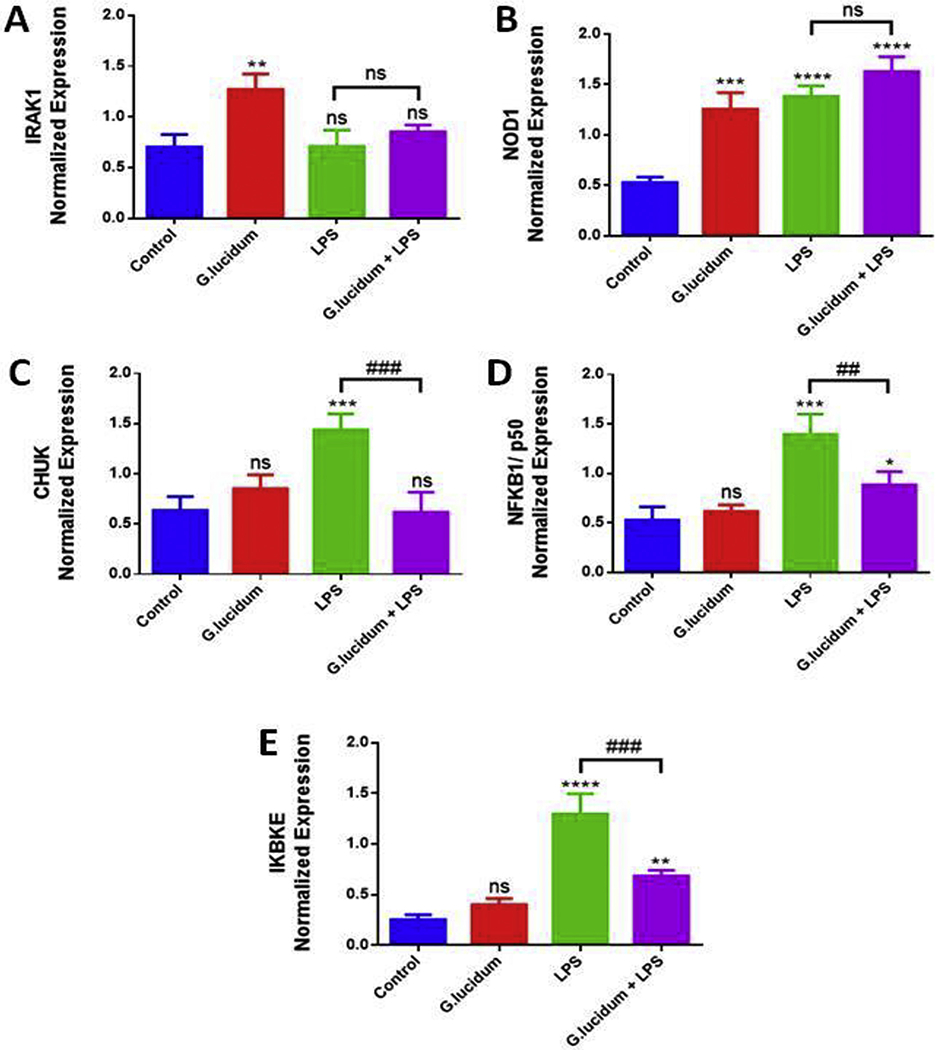
Down regulation of *IRAK1*, *NOD1*, *CHUK*, *NFKB1/p50*, and *IKBKE* mRNA expression by GLE pre-treatment in BV-2 microglial cells stimulated by LPS. Each data point represents the mean ± S.E.M. of three independent experiments (n = 3), representing 4 treatments: control (cells + dH_2_O), GLE (0.5 mg/ml) only, LPS (1 μg/ml) only, and GLE (0.5 mg/ml) + LPS (1 μg/ml) after 1 h, in a 24-h treatment period. Statistically significant differences between control vs treatments (*), and LPS vs GLE + LPS (^#^) were evaluated by a one-way ANOVA, followed by Dunnett’s multiple comparison tests. *p < .05, **p < .01, ***p < .001, ****p < .0001, ^##^p < .01, ^###^p < .001, ns = p > .05.

Quantitative real-time PCR was used to investigate the effect of GLE pre-treatment in genes associated with NFƙB and MAPK signaling pathways activation in BV-2 cells activated by LPS. The treatment with LPS significantly (*p* < .01) induced the mRNA expression of *NOD1*, *CHUK*, *NFƙB1/p50*, and *IKBKE* compared to the control, but not *IRAK1* (Fig. 5A–E). The increased fold-changes ranged from 2.2 to 5.2, with IKBKE presenting the highest increase in expression compared to the other genes (Fig. 5E). GLE pre-treatment significantly inhibited *CHUK*, *NFƙB1/p50*, and *IKBKE* expression (p < .01) in the LPS-stimulated BV-2 cells. GLE caused a 2-fold inhibition in the mRNA expression of these genes (Fig. 5C, D, and E), indicating that these genes may participate in the signaling pathway that leads to the release of the cytokines inhibited in the cytokine arrays and ELISA assays. GLE treatment by itself increased the expression of *IRAK1* (Fig. 5A) and *NOD1* (Fig. 5B), consequently, when cells were pre-treated with GLE and LPS (after 1 h), no statistically significant decrease in *IRAK1* and *NOD1* expression was detected, indicating no inhibitory effect of GLE over these genes.

## 5. Discussion

Neuroinflammation is a shared pathological feature in the onset and progression of several neurodegenerative diseases, including AD (Liu and Hong, 2003; Block and Hong, 2005; Chen et al., 2007; Gao and Hong, 2008; Kempuraj et al., 2016; Cobourne-Duval et al., 2018). While AD is characterized by neurofibrillary tangles and neuritic plaques, recent studies have implicated neuroinflammation as another culprit in disease progression (Tilstra et al., 2011, Mendonca et al., 2017; Mendonca et al., 2018, Mendonca et al., 2019,). Neuroinflammation is initially a protective mechanism, where inflammatory mediators work to restore damaged neuronal and glial cells during acute neuroinflammation (Azizi and Mirshafiey, 2012; Cobourne-Duval et al., 2018; Kempuraj et al., 2016; Von Bernhardi et al., 2015). However, chronic neuroinflammation tends to cause more neuronal damage and eventual degradation (Kempuraj et al., 2016; Leszek et al., 2016; Cobourne-Duval et al., 2018). NFƙB is involved in plaque formation in AD as well as in inflammation and cytokine signaling of AD progression (Mendonca et al., 2018; Tilstra et al., 2011). Studies have suggested that (Aβ) stimulation of NFƙB activity in microglia is one of the possible mechanisms for the increase in cytokines expression seen in AD (Combs et al., 2001; Mendonca et al., 2018). Suppression of NFƙB in microglia exhibits decreased neurotoxicity (Chen et al., 2016). MAPK signal transduction pathways, such as ERK, c-Jun N-terminal kinase (JNK), and p38, also play a role in immune-mediated neuroinflammatory responses and are implicated in the pathogenesis of AD (Hommes et al., 2003; Mendonca et al., 2018; Zhu et al., 2002). Finding therapeutic targets for these specific pathways could potentially decrease inflammatory responses and thus neurodegenerative disease progression.

In the current study, the effect of GLE was tested in LPS-nitrite production and in a broad range of 120 pro-inflammatory cytokines using mouse arrays to identify possible new targets to prevent neurodegenerative diseases. Our data indicate that pre-treatment with GLE decreased nitrite production in a dose-dependent manner, corroborating studies showing GLE effects on reducing acute inflammation in both RAW 264.7 macrophages (Hasnat et al., 2015) and BV-2 microglial cells (Yoon et al., 2013). Nitric oxide is a key mediator in several pathological processes, and its overproduction has a cytotoxic effect through the formation of peroxynitrite with superoxide anion. Woo et al. (2005) demonstrated that *G. lucidum* extracts suppressed LPS-induced iNOS mRNA expression and NO production in human monocytic cell-derived macrophages. This effect was mediated via its antioxidant action against LPS-induced superoxide anion generation in macrophages, suggesting that *G. lucidum* may exert a therapeutic effect against atherosclerosis via ameliorating iNOS-mediated NO overproduction in macrophages (Woo et al., 2005). Our results also showed that GLE pre-treatment inhibits the expression of the pro-inflammatory cytokines G-CSF, IL1a, MCP-5, MIP3α, and RANTES, which have not been described before. Pre-treatment with GLE was able to modulate the expression of these proteins, presenting a higher downregulation in MIP3α with a 13-fold-change decrease in expression, followed by RANTES with a 4.2-fold-change reduction in protein expression. Although LPS also upregulated the expression of CXCL1 and IL-6, GLE pre-treatment did not exert any inhibitory effect over these two cytokines.

GLE pre-treatment was very potent in the downregulation of MIP3α (also known as CC chemokine ligand 20/CCL20) expression. Together with CC chemokine receptor 6 (CCR6), MIP3α is recognized for its therapeutic potential in immunological research (Scheerens et al., 2001; Teramoto et al., 2005; Atreya and Neurath, 2010; Pezoldt and Huehn, 2016). The binding of CCL20 to CCR6 receptor controls immune homeostasis and activates immune response, presenting a high immunological impact in health and disease, and affecting multiple organs (Ranasinghe and Eri, 2018; Lee et al., 2015). The CCR6 and CCL20 axis has been demonstrated to directly influence the nervous system, as well as gastrointestinal, respiratory, excretory, and reproductive systems through immune mechanisms, leading to diseases with elevated mortality rates. Because of the critical role of CCR6 and CCL20 in clinical pathophysiology, this combination may be considered as a potential therapeutic target, and its inhibition or suppression may be a successful pharmacotherapeutic treatment for its associated diseases (Proudfoot, 2002; Comerford et al., 2010; Ito et al., 2011).

Furthermore, in our studies, pre-treatment with GLE decreased the expression of RANTES/CCL5, whose downregulation may help to ameliorate inflammatory stages since it has been described in acute inflammation as a chemoattractant that recruit’s leukocytes into inflammatory sites (Cobourne-Duval et al., 2018). It also mediates the chemotaxis of microglia toward Aβ aggregates characteristic of AD neuropathology (Huang et al., 2009; Cobourne-Duval et al., 2018;). The microglial clustering around neuritic plaques contribute to neuroinflammation and progressive neurodegeneration, and CCL5 down-regulation reduces chemotaxis of microglia toward Aβ aggregates (Huang et al., 2010; Cobourne-Duval et al., 2018). Recent studies in our laboratory have shown that treatment of BV-2 microglial cells with thymoquinone, an active component of *Nigella sativa* seed oil, decreased gene expression of RANTES/CCL5 by 7-fold compared to untreated cells (Cobourne-Duval et al., 2018). Also, pristimerin, a naturally occurring triterpenoid with antitumor and anti-inflammatory activities, has been shown to significantly suppress the release of RANTES in LPS-activated BV-2 microglial cells (Hui et al., 2018). Conversely, RANTES has also been implicated as being neuroprotective. Treatment of neurons in vitro with RANTES results in an increase in cell survival and a neuroprotective effect against the toxicity of thrombin and sodium nitroprusside (Tripathy et al., 2010; Liu et al., 2014,). RANTES is also up-regulated in the substantia nigra of mouse models of PD, and the neutralization of RANTES protects against nigrostriatal degeneration (Chandra et al., 2016; Cobourne-Duval et al., 2018).

Although we used a crude extract containing a mix of phytochemical constituents, our results indicate that pre-treatment with GLE has a great potential to attenuate the expression of different pro-inflammatory cytokines whose overexpression is involved in neuroinflammation and neurodegenerative diseases. Cytokines mediate the rapid activation of NFƙB through activation of the IKK complex, which contains CHUK and IKBKE, leading to subsequent phosphorylation and degradation of the inhibitory IκB proteins (Adli et al., 2010; Mendonca et al., 2018,). Phosphorylation of IκB by the IKK complex involves kinases such as NFƙB1/p50 which is a crucial step in NFƙB activation, subsequent phosphorylation, and nuclear translocation of NFƙB1 dimers, and transcription of target genes (Zandi et al., 1997; Gilmore, 2006, Scheidereit, 2006, Cobourne-Duval et al., 2018,). In the present study, possible signaling pathways involved in the GLE inhibitory effect over the release of cytokines were investigated. GLE pre-treatment modulated the expression of different genes associated, directly or indirectly, to NFƙB, MAPK, and NOD activation. Our data show that pre-treatment with GLE significantly downregulated the expression of *CHUK*, *NFKB1/p50*, and *IKBKE*, all of which are involved in the signaling cascades that activate NFƙB and MAPK pathways, leading to inflammatory cytokines production. On the contrary, GLE alone increased the expression of *IRAK1* and *NOD1*, having no inhibitory effect on the expression of these genes, and indicating that GLE does not affect NOD pathway targeting NOD1, which is a member of NOD-like receptor protein family. LPS binding to NOD-like or toll-like receptors promotes inflammation, activating NFƙB and MAPK signaling pathways, which triggers the transcription of genes and translation of proteins involved in this inflammatory response (Farzi et al., 2015). Therefore, our results suggest that GLE pre-treatment may modulate the activation of NFƙB and MAPK signaling pathways by downregulating the expression of critical genes involved in the pro-inflammatory process, and it seems that this modulation happens via the toll-like receptor pathway genes, but may not affect genes of the NOD-like receptor signaling pathway.

## 6. Conclusion

The data clearly show that pre-treatment with GLE extract inhibits the expression of cytokines and chemokines in microglia cells stimulated by LPS, mainly reducing the expression of RANTES and suppressing the expression of MIP3α almost completely. Results also demonstrate the pre-treatment with GLE decreases gene expression of *CHUK*, NFƙB*1/p50*, and *IKBKE*, which may be associated with the etiology and neuropathology of AD through activation of NFƙB and MAPK signaling pathways. The pre-treatment with GLE extract was able to reduce the expression of RANTES and to block almost 100% the expression of MIP3α. Since these proteins contribute to neuroinflammation and progression of neurodegeneration, we believe that GLE extract may have a potential role in the prevention of neurodegenerative diseases. Future studies with individual phytochemical constituents of *G. lucidum* are needed, as well as animal models of neurodegenerative diseases to substantiate GLE clinical utilization for the prevention of these diseases.

## Abbreviations:

Aβ: Amyloid Beta
AD: Alzheimer’s disease
AKT: Protein kinase B
CCL20: CC chemokine ligand 20
CCR6: CC chemokine receptor 6
CHUK: Conserved helix-loop-helix ubiquitous kinase
CXCL1: C-X-C Motif Chemokine Ligand 1
DeGA F: Deacetyl ganoderic acid F
ELISA: Enzyme-linked immunosorbent assay
ERK: Extracellular signal-regulated kinase
G-CSF: Granulocyte-colony stimulating factor
GLE: *Ganoderma lucidum* extract
IKBKE: Inhibitor of nuclear factor kappa-B kinase subunit epsilon
IKBα: nuclear factor of kappa light polypeptide gene enhancer in B-cells inhibitor, alpha
IKKα/β: inhibitor of nuclear factor kappa-B kinase subunit alpha/beta
IL1α: Interleukin 1 alpha
IL-1β: Interleukin 1 beta
IL-6: Interleukin 6
iNOS: Inducible nitric oxide synthase
IRAK1: Interleukin 1 receptor-associated kinase 1
LPS: Lipopolysaccharides
MAPK: Mitogen-activated protein kinase
MCP-5: Murine monocyte chemoattractant protein-5
MIP3α: Macrophage inflammatory protein-3α
NFKB1: Nuclear factor-kappa B subunit 1
NFƙB: Nuclear factor kappa-light-chain-enhancer of activated B cells
NO: Nitric oxide
NOD1: Nucleotide-binding oligomerization domain-containing protein 1
PD: Parkinson’s disease
PM: Primary Microglia
RANTES: Regulated on activation, normal T cell expressed and secreted
TGFβ: Transforming growth factor-beta
TNF-α: Tumor necrosis factor-alpha
